# Old and Young Actors Playing Novel Roles in the Drama of Multiple Myeloma Bone Marrow Microenvironment Dependent Drug Resistance

**DOI:** 10.3390/ijms19051512

**Published:** 2018-05-18

**Authors:** Sabrina Manni, Marilena Carrino, Gianpietro Semenzato, Francesco Piazza

**Affiliations:** 1 Department of Medicine, Hematology Section, University of Padova, Via N.Giustiniani 2, 35128 Padova, Italy; marilena.carrino@studenti.unipd.it (M.C.); g.semenzato@unipd.it (G.S.); 2 Venetian Institute of Molecular Medicine, Via G.Orus 2, 35129 Padova, Italy

**Keywords:** multiple myeloma, bone marrow microenvironment, drug resistance

## Abstract

Multiple myeloma (MM) is the second most frequent hematologic cancer. In addition to the deleterious effects of neoplastic plasma cell growth and spreading during the disease evolution, this tumor is characterized by the serious pathological consequences due to the massive secretion of monoclonal immunoglobulins and by the derangement of bone physiology with progressive weakening of the skeleton. Despite significant progresses having been made in the last two decades in the therapeutic management of this plasma cell tumor, MM remains invariably lethal, due to its extremely complex genetic architecture and to the constant protection it receives from the tumor niche, which is represented by the bone marrow microenvironment. While it is predictable that the discovery of novel therapies against the first of these two pathobiological features will take a longer time, the identification of the cellular and molecular mechanisms underlying the pro-growth effects of the myeloma milieu is a task that could lead to the development of novel treatments in a shorter timeframe. In this regard, aside from known “old” determinants of the cross-talk between bone marrow and MM cells, “young” cellular and molecular factors are now emerging, taking the scene of this complex neoplastic setting. In this review we aimed at giving insights on the latest evidence of potentially-targetable modes that MM cells exploit to increase fitness and gain a survival advantage. The benefits coming from the derangements of stress-managing pathways, autophagy, transcriptional rewiring, and non-coding RNAs are examples of such methods that MM cells utilize to escape cell death, but that hopefully will offer novel targets for the ever-increasing anti-MM therapeutic armamentarium.

## 1. Introduction

Multiple myeloma (MM) is a plasma cell neoplasia representing 10–15% of all the hematological malignancies. Significant progress has been made in the last two decades regarding the comprehension of MM pathogenesis, as well as the development of novel, more effective therapies, such as proteasome inhibitors and immunomodulatory drugs (IMiDs) [1]. Exciting progress has also been achieved in the field of immunotherapy in MM. The recognition of CD38, CD319/signaling lymphocytic activation molecule F7 (SLAMF7), and B-cell maturation antigen (BCMA) as targetable surface-expressed molecules on malignant plasma cells has led to the development of monoclonal antibodies, such as daratumumab (anti-CD38), elotuzumab (anti-CD319), and anti-BCMA, which are now available to the clinician for use in the first line and relapsed/refractory setting [2]. BCMA-targeting Chimeric Antigen Receptor (CAR)-T cells are another striking example of active cancer immunotherapy that is currently tested in MM, and results from early studies are encouraging [3]. Likely, immunotherapy will also enter the therapeutic arena soon in MM [4]. However, MM remains an almost invariably fatal disease endowed with a great burden of morbidity due to the MM-associated bone disease, renal insufficiency, and susceptibility to infections and bone marrow failure. Therefore, it is important to develop novel therapeutic options acting against the malignant plasma cellular clones, as well as against the pro-myeloma mechanisms in the bone marrow (BM) microenvironment. Indeed, the role of the BM microenvironment in sustaining the disease is well established [5]. Modifications occurring in the malignant BM milieu may mediate MM cell survival and resistance to chemotherapy through neoangiogenesis, the establishment of immunosuppression, cell-cell interactions with blood cells, vascular endothelial cells, osteoclasts, osteoblasts, stromal cells, adipocytes [6,7], the delivery of soluble factors, such as cytokines and chemokines [8] and by metabolic changes, such as hypoxia or nutrient deprivation [9]. Moreover, new players and mechanisms have also recently emerged as active BM microenvironment-associated participants in MM pathogenesis: examples are upregulation of stress managing pathways, the dysregulation of non-coding RNA species, such as miRNA, the release of cellular components, such as exosomes or microvescicles, and other cellular populations, like Cancer Associated Fibroblasts (CAFs) [10]. 

All of these components and modifications of the BM microenvironment are known to orchestrate a fine tuning of signaling pathways that ultimately favor plasma cell homing, survival, and proliferation [7]. Many drugs that target the BM milieu have been demonstrated to possess a therapeutic efficacy in MM preclinical models and some are currently being tested in clinical trials [11].

The present review will focus on new mechanisms/features peculiar of MM plasma cells (PCs) that could sustain BM-mediated drug resistance and that could be therapeutically explored as novel ways of treating this “hard to beat” disease.

## 2. Stress Pathways and Malignant Plasma Cell Fitness

The physiological development of PCs is characterized by the activation of stress pathways and by the consequent dependency on cellular mechanisms acting as stress managers [12]. For instance, the acquisition of a secretory phenotype by terminally-differentiated B cells to ensure the production of antibodies and the humoral immune response needs a reprogramming of the mechanisms dedicated to the synthesis of organelles (endoplasmic reticulum, vesicles, mitochondria) to the incoming massive protein synthesis, as well as the activation of compensatory methods to clear up abnormal proteins and organelles, such as the ubiquitin proteasome system (UPS) and autophagy [12,13,14]. Malignant PCs display a hypertrophy of these mechanisms. The result is the upregulation of specific signaling pathways of stress-coping that could have a two-fold consequence on MM cells: on one hand, they cause the potentiation of anti-apoptotic cascades that may contribute to the growth of the malignant clones; on the other hand, they generate vulnerabilities that can be targeted for therapeutic purposes [15]. In addition to intrinsic triggers of stress, cancer cells receive stress signals from the bone marrow microenvironment, like hypoxia, reactive oxygen species (ROS), extracellular paraprotein, low glucose content, etc. All these stimuli may converge in eliciting intracellular stress pathways [16].

### 2.1. The Endoplasmic Reticulum Stress/Unfolded Protein Response: A Life Vest for MM Cells to Escape from Chemotherapeutic Agents

The role of the endoplasmic reticulum (ER) stress/unfolded protein response (UPR) in the pathogenesis of MM of malignant PCs has been demonstrated by several studies [17,18,19]. The ER stress is caused by the accumulation of misfolded/unfolded proteins in the ER lumen at a rate exceeding the compensatory mechanisms of refolding or degradation. Irregular proteins may accumulate because of overload (especially in secretory cells, like PCs) of altered transcription/translation, mutations in coding genes, or due to damaging stresses, such as oxidative stress, chemotherapeutic agents, or ionizing radiation [20,21]. The UPR is a complex cellular response to ER stress. Three main sensors of the ER stress are known: the kinase/endoribonuclease inositol, requiring enzyme 1 (IRE1α); the kinase PKR-like Endoplasmic Reticulum kinase (PERK); and the transcription factor activating transcription factor-6 (ATF6). In conditions of homoeostasis, these proteins are bound to the molecule Glucose-regulated protein 78/Binding immunoglobulin protein (Grp78/Bip) that inhibits their activity. With the progressive accumulation of misfolded/unfolded proteins in the ER, the probability that hydrophobic stretches interact with Grp78/Bip increases and Grp78/Bip is attracted away from the three sensors that are then unleashed and become active. IRE1α’s main function is to cut a 26 base pair intron from the mRNA of the transcription factor X-box Binding Protein 1 (XBP1) so that a longer open reading frame is created, coding for the active form of the transcription factor [22]. XBP1 spliced (XBP1s) is a potent transcription factor (TF) that activates the transcription of targets belonging to a group of stress-managing genes, such as foldases, oxido-reductases, intracellular trafficking and protein import pathways, autophagy, chaperones, ER membrane biosynthesis, ER-associated degradation (ERAD) components, and glycosylases [17,23]. XBP1 also upregulates pro-inflammatory cytokine production, lipid biosynthesis, and hypoxia-related genes [24]. The increased cellular levels of many of these effectors facilitates the setting up of compensatory mechanisms able to manage the ER stress. However, upon extreme ER stress, IRE1α oligomerize lowering some ER-resident mRNA and microRNA important for cell survival, in a proapoptotic manner through the so-called regulated IRE1α-dependent decay process (RIDD) [25,26].

The activation of PERK causes the phosphorylation of the eukaryotic translation initiation factor 2α (eIF2α) with consequent inhibition of its function and of protein translation, except for some proteins, such as the transcription factor 4 (ATF4) and the nuclear factor-erythroid 2–related factor 2 (Nrf2) [27]. The global functional significance of the PERK axis is to lower protein translation with the consequent reduction of the global protein load in the ER, thus allowing refolding activity of ER-resident chaperones. However, the selective translation of ATF4, directly upregulates the transcription of the proapoptotic gene C/EBP-homologous protein CHOP, which cooperates with ATF4 and Nrf2 in inducing a transcriptional anti-stress response [16]. The ATF6 arm of the UPR activates also a transcriptional response aimed at managing ER stress. ATF6 is released from the ER through an intermediate step in the Golgi apparatus by site 1 and site 2 protease. Its DNA-binding domain enters the nucleus and activates a transcriptional response that increases the levels of Bip/Grp98 and a subset of XPB1-dependent chaperones, oxidoreductases, quality control, and ERAD machinery [23]. These proteins serve as anti-stress response effectors. MM cells are exquisitely dependent on the UPR since they are chronically exposed to a considerable proteotoxic stress, which results from the sum of the large burden of Ig synthesis and of the accumulation of misfolded polypeptides due to the malignant phenotype. Therefore, MM cells are particularly sensitive to perturbations of the UPR and this has been the rationale of the efficacy of the proteasome inhibitors (PIs), like the first in class bortezomib and second-generation carfilzomib, ixazomib, or marizomib, in this disease. However, resistance to PIs may emerge leading to refractoriness and progression of MM.

Evidence that stress pathways may confer resistance to current and novel anti-MM therapies has also been documented. The resistance to chemotherapeutic and other cytotoxic agents may derive from the rewiring of the cellular phenotype due to the chronic activity of the stress pathways [28]. For instance, it has been shown that overexpression of Grp78/Bip may confer chemoresistance and worse prognosis in cancer patients [29,30,31]. XBP1s is involved in adaptation to hypoxia, which leads to increased misfolded protein levels, activating IRE1α [32]. Moreover, among the kinome, IRE1α mutations are the most recurrently found in cancer [33]. XBP1 seems to play a pivotal role in the pathogenesis of MM [34]; its expression is often high in MM [35], and mutated XBP1 has been observed in MM and other cancer patients [36].

**Figure 1:**
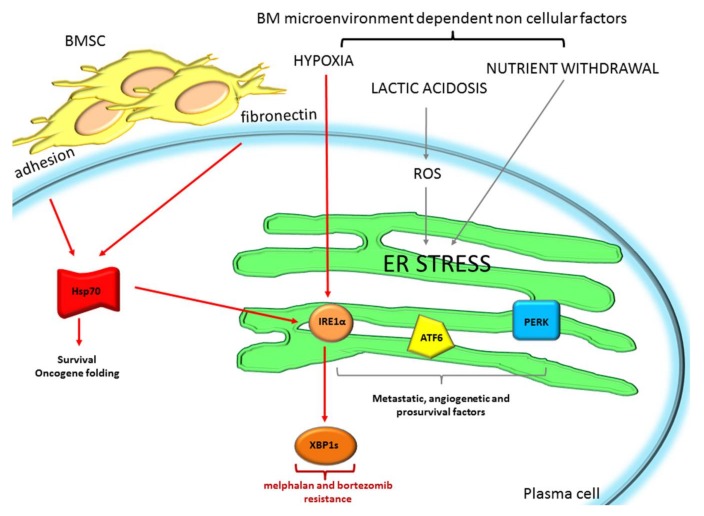
Bone marrow (BM) microenvironment cells and non-cellular components promote protective Endoplasmic reticulum (ER) stress mediating drug resistance. In red are shown cellular (Bone Marrow Stromal Cells (BMSC) adhesion) and non-cellular factors (fibronectin and hypoxia), that activate protective ER stress in multiple myeloma (MM), promoting MM drug resistance to melphalan and bortezomib. In grey are depicted general cancer mechanisms mediated by other non-cellular BM microenvironment components (lactic acidosis and nutrient withdrawal) that could also be potentially involved in MM drug resistance, promoting metastasis, angiogenesis, and plasma cell (PC) survival. ROS: Reactive Oxygen Species; Hsp70: Heat Shock Protein 70; IRE1α: Inositol Requiring Enzyme 1α; ATF6: Activating Transcription Factor-6; PERK: kinase PKR-like ER kinase; XBP1s: X-box Binding Protein 1.

Heat shock chaperone proteins (HSPs) are particularly important for protein quality control. They support MM cell survival and proliferation maintaining the proper folding of several oncogenes or newly synthetized proteins [37]. It has recently been reported that Hsp70 mediates drug resistance and it is an important survival promoter in MM, protecting MM cells from ER stress-mediated cell death, through the increased splicing of XBP1 [38,39]. The BM microenvironment sustains MM cell survival by impinging on ER stress/UPR dependent programs. Attachment of MM cells to bone marrow stromal cells (BMSCs) or fibronectin could be responsible of melphalan and bortezomib resistance through the upregulation of Hsp70, and subsequently enhanced the protection of XPB1 levels [39,40] (Figure 1).

Moreover, in cancer cells, the BM microenvironment can induce ER stress, through oxygen and nutrient withdrawal and lactic acidosis that could increase ROS production [16]. All these factors could limit cancer cell ER protein folding, causing chaperone dysregulation, and enhancing ER stress/UPR. The activation of UPR through all its three branches ultimately sustains malignant cell progression, through the subsequent activation of intra- or extracellular factors involved in metastasis (Hypoxia-inducible factor 1α HIF1α, Nrf2), angiogenesis (Vascular Endothelial Growth Factor VEGF, Interleukin-8 IL-8, Fibroblast Growth Factor 2 FGF2, formation of new vessels), and PC growth (HIF1α, Nrf2, Nuclear Factor kappa-light-chain-enhancer of activated B cells NF-κB, autophagy) that could help in coping with difficult BM microenvironment conditions. In particular, the protective role of the IRE1α/XBP1 branch of the UPR is demonstrated in the hypoxic niche of the BM microenvironment in MM. MM cells grown in the hypoxic microenvironment activates IRE1α with augmented XBP1 splicing [41], which may be responsible for drug resistance (Figure 1). Indeed, IRE1α endonuclease inhibitors such as toyocamycin, STF-083010 4μ8C, MKC-3946, and B-I09, which prevent XBP1 splicing, have been successfully exploited in MM [41,42], and other cancers [43] and could be considered as a therapeutic strategy to diminish cancer cell ability to live in harsh microenvironment conditions, such as hypoxia and nutrient deprivation, leading to increased drug sensitivity and reduced angiogenesis.

In MM, other several mechanisms may occur to potentially contribute the chemoresistant phenotype. For instance, Grp78/Bip overexpression [29], PERK/Nrf2 axis upregulation with increased levels of anti-oxidant proteins, such as heme oxygenase 1 [44,45], increased intracellular levels of glutathione [46], or of protein-degrading proteasome maturation proteins (POMP) [47] are processes that may participate in conferring resistance to chemotherapeutics, as well as proteasome inhibitors. Another intriguing way MM cells may resist proteasome inhibitor-induced cell death is through the maintenance of a pool of less-differentiated CD20^+^ MM cell precursors that display a hyposecretive phenotype and low levels of XBP1s [48]. 

Moreover, our laboratory has identified protein kinase CK2 as a critical regulator of ER stress/UPR in MM, impacting the proteotoxic/UPR stress [49,50]. CK2 is a pleiotropic protein kinase which sustains malignant PC growth and proliferation by different means including, among others, the regulation of important prosurvival signaling cascades, such as NF-κB and STAT3, and PC stress copying functions. Indeed, CK2 maintains the ER stress homeostasis in MM by mainly regulating IRE1α and PERK axes of the UPR. Moreover, we demonstrated that the combined treatment of MM cells with a CK2 inhibitor together with ER stressor agents, such as thapsigargin and geldanamycin, caused a synergistic cytotoxic effect on MM cells cultured alone or on stromal cells in a BM microenvironment model, suggesting a strong role of CK2 in ER stress-mediated MM cell survival, also in the context of BM-induced cell protection.

### 2.2. Autophagy Can Sustain Drug Resistance in MM

Autophagy is an evolutionarily-conserved and highly-regulated catabolic process that mammalian cells could activate to react to adverse environmental derangements [51]. In MM PCs, autophagy, through the recycling of cellular organelles and of accumulating misfolded proteins may relieve the ER stress, avoiding excess proteotoxicity, and ensuring cell survival.

It has been demonstrated that autophagy must be tightly controlled in normal and malignant PCs.

B cells of mouse models unable to activate autophagy due to the deletion of the protein Atrogin 5 (Atg5) were shown to be able to differentiate in vitro in PCs. However, these PCs showed a more expanded ER and greater antibody production than PCs differentiated from wild-type B cells, suggesting that autophagy regulates ER homeostasis. Moreover, in the BM of *Atg5* KO mice, only PCs that did not delete the *Atg5* gene (due to an inefficient CRE-mediated recombination, a well-known phenomenon) were found, demonstrating that autophagy is required for long-lived PC establishment [14]. Thus, it is not surprising that deregulation of the autophagic process is maladaptive in MM. It has been demonstrated that impairment of autophagy causes MM cell apoptosis and, oppositely, an excess of autophagy has also a negative impact on MM cell growth. The silencing of autophagy essential genes, such as *Atrogin 7* (*Atg7*) or *Sequestosome-1/p62* (*SQSTM1/p62*), affects MM cell survival and, in particular, silencing of *SQSTM1/p62*, together with bortezomib treatment, enhanced bortezomib-dependent toxicity and ubiquitinated protein accumulation [52]. Nevertheless it was shown that also uncontrolled autophagy can reduce MM cell survival. Lamy et al., demonstrated that caspase-10 is essential for MM viability, since its depletion caused hyperactive autophagy that terminates in cell death in the absence of the hallmarks of apoptosis [53].

Different studies were performed in order to analyze the effects of anti-MM drugs on autophagy and modulation of autophagy is being explored as a rational strategy to enhance MM cell killing. However, results have so far been heterogeneous. For instance, thapsigargin (an ER stress inducer that also causes autophagy in MM) synergized with the autophagic inhibitor 3-methyladenine (3-MA) in inducing MM cells cytotoxicity [54] and apoptosis was strongly induced in MM cells treated with carfilzomib (a second-generation proteasome inhibitor that induces compensatory autophagy) together with the autophagic inhibitor chloroquine (CQ) [55]. Oppositely, the combination of bortezomib (a proteasome inhibitor that increased basal autophagy in MM cells) with both 3-MA or CQ, reduced bortezomib-induced apoptosis, suggesting an antagonistic effect of autophagy inhibitors and bortezomib [54]. Therefore, further experiments will be needed to better clarify the role of autophagy upon proteasome inhibition.

**Figure 2:**
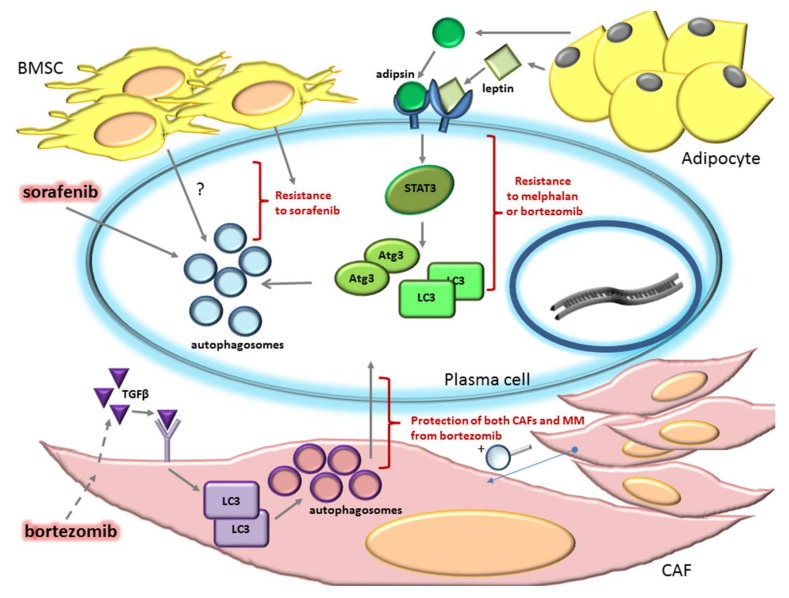
Autophagy can promote bone marrow (BM) microenvironment dependent drug resistance in multiple myeloma (MM). BM microenvironment cells confer resistance to MM plasma cells (PCs) against different drugs such as sorafenib, melphalan, and bortezomib, through Bone Marrow Stromal Cells (BMSCs) and adipocytes induced autophagy activation. Autophagy can also play a protective role in Cancer Associated Fibroblasts (CAFs). Activation of autophagy through CAFs-secreted Tumor Growth Factor-β (TGFβ) mediates CAFs and PC resistance to bortezomib. Dashed arrows indicate the bortezomib-induced release of TGFβ by CAFs. STAT3: Signal transducer and activator of transcription 3; Atg3: Atrogin 3; LC3: microtubule-associated protein 1A/1B-light chain 3.

Nevertheless, autophagy seems to be a survival mechanism that PCs and other microenvironmental cellular components activate in response to several anti-MM drugs (Figure 2). For instance, it has been found that autophagy protects MM cells from the cytotoxic effect induced by Sorafenib (Sor), a multiple tyrosine kinase inhibitor commonly used in renal and hepatocellular carcinoma [56], and a promising agent also for MM. In fact, Sor inhibits multiple tyrosine kinases that may be active in MM cells, playing an important role in the maintenance of MM survival and proliferation [57]. Kharaziha et al. [58] showed that Sor caused MM cell death, but also induced autophagy in MM cells, as demonstrated by reduced activity of the negative regulator of autophagy mTOR, by p62 degradation, and by microtubule-associated protein 1A/1B-light chain 3 (LC3) lipidation (LC3 II). However, Sor-induced autophagy could be an escaping mechanism of MM cells from apoptosis, since the inhibition of autophagy with 3-MA or CQ enhanced Sor-induced MM cell death, whereas the induction of autophagy with Rapamycin reduced Sor-dependent cytotoxicity. Moreover, a reduction in the sensitivity to Sor was observed in MM cell lines and patient derived PCs when co-cultured with the BMSC cell line L88 and it has been speculated that a possible mechanism of resistance to Sor could be the induction of autophagy by the tumor microenvironment [58,59]. Indeed, the BM microenvironment induced a protective autophagy in malignant PCs through adipocyte-secreted adipokines, such as adipsin and leptin [60]. These adipokines activate STAT3, with the consequent expression of autophagic proteins, such as Atg3 and LC3. Adipocyte-induced autophagy protects myeloma cells from chemotherapeutics, as judged by the fact that, in co-cultures of MM cells with adipocytes or with adipocyte-derived culture media, the rate of apoptosis of MM cell lines treated with melphalan or bortezomib was significantly enhanced by the concurrent use of the autophagy inhibitors 3-MA or CQ, therefore suggesting an essential role of autophagy in adipocytes-mediated drug resistance [60].

A role of autophagy has also been demonstrated in the dynamics of cell-cell interaction between MM cells and CAFs, another cancer type in the stroma that protects MM PCs from drugs, including bortezomib. The most important environmental stimulus responsible for the conversion of normal fibroblasts into CAFs is Tumor Growth Factor-β (TGFβ) [61]. In the study of Frassanito et al. [62] it was shown that exposure to bortezomib of MM patient CAFs promotes the secretion of cytokines, including TGFβ. TGFβ stimulation or bortezomib treatment of CAFs, not only increased phosphorylation of the TGFβ downstream target Small mothers against decapentaplegic 2/3 (Smad2/3), but also enhanced the autophagic pathway, as demonstrated by increases in LC3-II expression. Moreover, CAFs collected from bortezomib-resistant patients show intrinsic activation of autophagy and their treatment with the autophagic inhibitor 3-MA, restoring their sensitivity to bortezomib, suggesting a pro-survival role of autophagy in CAFs. Interestingly, treatment of bortezomib-resistant CAFs with TGFβ inhibitors together with bortezomib reduces autophagy and increases apoptosis in fibroblasts, with the consequent impairment of their ability to protect PCs from bortezomib, thus, rendering them susceptible to this drug [62].

In conclusion, the ER stress/UPR and autophagy are essential life vests for MM cells that can mediate MM drug resistance. Therefore, targeting some key players of this game could have beneficial therapeutic effects.

## 3. The Bone Marrow Microenvironment Dependent Modulation of Gene Expression Induces Drug Resistance in Plasma Cells

**Figure 3:**
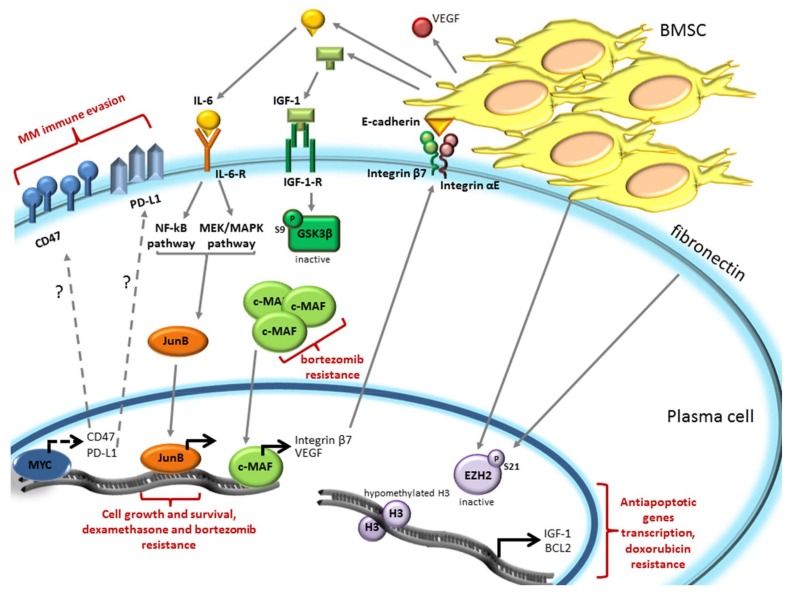
Modulation of the BM microenvironment dependent drug resistance by V-Myc Avian Myelocytomatosis Viral Oncogene (MYC), JunB, Avian Musculoaponeurotic Fibrosarcoma (c-MAF) transcription factors, and by the Enhancer of zeste homolog 2 (EZH2) methyl transferase-mediated epigenetic control of histone H3. MYC-dependent CD47 and Programmed Death Ligand-1 (PD-L1) expression promotes cancer immune evasion. Dashed lines indicate that this phenomenon could be present also in MM. BMSCs secreted Interleukin-6 (IL-6) and Insuline-like Growth Factor 1 (IGF-1) mediate drug resistance through the activation of JunB and c-MAF TFs. cMAF, in turn, establishes an activatory feedback loop whereby the BM microenvironment milieu is ameliorated through the upregulation of cMAF-dependent transcription of integrin B7 and Vascular Endothelial Growth Factor (VEGF). BMSC or fibronectin promotes doxorubicin resistance by enhancing the inactivating phosphorylation of Ser 21 in EZH2, leading to the transcription of anti-apoptotic genes, such as *IGF-1* and B-cell lymphoma 2 (*Bcl2*).

The BM microenvironment was shown to modulate PC gene expression through the regulation of pivotal TFs that promote drugs resistance. In particular, V-Myc Avian Myelocytomatosis Viral Oncogene (MYC), JunB and Avian Musculoaponeurotic Fibrosarcoma (c-MAF) TFs, and the methyl transferase Enhancer of Zeste Homolog 2 (EZH2) play an emerging role in immune evasion and drug resistance (Figure 3).

MYC is an oncogenic protein that acts as a transcription factor. Among the many processes this protein regulates, a peculiar role seems to be its involvement in MM immune evasion. The MYC signature is not expressed in normal and Monoclonal Gammopathy of Undetermined Significance (MGUS) PCs, whereas it is found in 67% of MM patients, suggesting that MYC activation is a central event in the progression of the disease [63,64]. Overexpression of MYC occurs as a consequence of the translocation of coding gene in the Ig locus or on some non-Ig partner, such as *Family with Sequence Similarity 46 Member C* (*FAM46C*), *Forkhead Box O3* (*FOXO3*), and *Bone Morphogenetic Protein 6* (*BMP6*) that put the *MYC* gene under the control of enhancers or super-enhancers [65,66,67]. MYC translocations are frequent in newly-diagnosed MM, but are rarely detected in MGUS and smoldering myeloma (sMM) patients (only in 3–4%) [68,69]. *MYC* abnormalities are significant risk factors for disease progression in MM. Moreover, *c-MYC* could be upregulated in MM cells by the autocrine or paracrine (BMSC-mediated) production of IL-6, which activates anti-apoptotic proteins, such as Myeloid cell leukemia 1 (Mcl1) and B-cell lymphoma-extra large (Bcl-XL), including c-MYC [8,70]. MYC controls several functions in cancer cells, such as inducing DNA replication, regulating splicing factors, activating ribosome biogenesis, and protein synthesis, and it is involved in tumor immune escape [68]. MYC could also sustain MM immune evasion by upregulating the expression of the innate regulator cluster of differentiation CD47 and of the adaptive immune checkpoint Programmed Death-Ligand 1 (PD-L1) on the cell surface [68,71]. Cancer cells in a Tet-inducible transgenic mouse model of MYC-induced T cell acute lymphoblastic leukemia (MYC T-ALL) and of hepatocellular carcinoma (HCC) expressed both CD47 and PD-L1 when MYC was turned on and, oppositely, the knockdown of MYC in several cancer cell lines (T-ALL, HCC, melanoma, and non-small cell lung cancer) caused a reduction in their expression [71]. The inactivation of MYC in tumor models results in recruitment of immune cells promoting tumor regression; conversely, the constitutive expression of CD47 and PD-L1 in MYC T-ALL mouse prevented tumor regression even in the absence of MYC [71]. Since MYC is upregulated in MM and, among others, enhances PD-L1 and CD47 membrane translocation in cancer cells, it is possible that MYC also regulates PD-L1 and CD47 expression in MM cells. This hypothesis has not yet been validated. However, in order to impair MM interconnection with the immune microenvironment anti-PD-L1 antibodies (among others atezolizumab and durvalumab) are being tested in clinical trials in MM patients [72,73].

Another TF recently demonstrated to mediate MM drugs resistance is JunB, a member of the Activator Protein 1 (AP-1) family of TF [74]. Its expression at the mRNA level progressively increased from normal to MGUS, sMM, and MM patient PCs, suggesting a correlation between JunB expression and MM pathogenesis. JunB expression in MM cells is mediated by soluble factors secreted by BMSCs and in particular by IL-6. IL-6, via Mitogen-activated protein kinase kinase/ mitogen-activated protein kinase (MEK/MAPK) and NF-κB pathways, triggers JunB upregulation in a time- and dose-dependent manner. Moreover, the use of tocilizumab (a humanized monoclonal IL-6 receptor antibody) prevents BMSC-dependent JunB upregulation. It was demonstrated that JunB supports MM cells growth and survival since shRNA-mediated *JunB* knockdown in MM cells co-cultured with primary BMSCs or stromal cell lines, inhibited BMSC-induced PC proliferation and increased apoptosis. Modulation of JunB expression was demonstrated to affect drug resistance or sensitivity. The glucocorticoid (GC)-resistant MM.1R cell line shows a higher basal level of JunB mRNA compared to GC-sensitive MM.1S cells. Silencing of *JunB* sensitized MM.1R cells to dexamethasone, inducing a reduction in cell proliferation and survival. Conversely, expression of a fusion protein of JunB with the hormone-binding domain of the human estrogen receptor (JunB-ER) that become active in the presence of 4-OHT (4-hydroxytamoxifen), revealed that 4-OHT treatment enhanced dexamethasone and bortezomib resistance of MM.1S cells. Therefore, the BM microenviroment, through IL-6 secretion, enhances JunB expression in MM cells promoting MM cell proliferation and conferring chemoresistance [74].

c-MAF is a member of the basic leucine zipper transcription factors belonging to the AP-1 superfamily and regulates gene transcription by cyclic adenosine monophosphate-response element [75]. *c-MAF* mRNA is highly expressed in t(14;16) translocated MM, in which *c-MAF* gene fused with the immunoglobulin heavy chain [76,77]. Even if this translocation is present only in 5–10% of MM patients, *c-MAF* results overexpressed approximately in 50% of MM cases [78]. Interestingly, patients carrying a *MAF* translocation respond poorly to proteasome inhibitors and high MAF protein levels conferred innate resistance to bortezomib [79,80,81]. The specific silencing of MAF protein in RPMI-8226 and ANBL6 cells (both harboring t(14;16) translocation) increased bortezomib response, while MAF overexpression in XG1 cells (lacking t(14;16) translocation) led to decreased bortezomib sensitivity. In addition, c-MAF protein stability was shown to be controlled by Glycogen synthase kinase 3β (GSK3β), as previously demonstrated for another member of the family, MAF-A [82]. GSK3β, by phosphorylating c-MAF in T58-62, induced its proteasomal degradation [83]. GSK3β inhibition with SB216763 in MM144 cells [79] and with LiCl in RPMI-8226 [83] or proteasome inhibition with bortezomib [79] resulted in c-MAF accumulation. BMSCs secreted IGF-1, by inactivating GSK3β through the phosphorylation of Ser9, prevents GSK3β-dependent phosphorylations of c-MAF leading to its accumulation, therefore, enhancing bortezomib resistance **[79]**. c-MAF is also important for BMSCs-MM cell interaction. Indeed, c-MAF induces integrin β7 expression, an adhesion molecule that heterodimerizes with integrin αE to bind E-cadherin on BMSC surface. This interaction significantly increases the secretion of VEGF to ameliorate the microenvironment protection and its angiogenetic proprieties [78]. 

A novel mechanism of BM induced drug resistance was shown by Kikuchi et al. [84]. It involved the epigenetic control of the target of the methyl transferase EZH2, histone H3, which, when trimethylated in Lysine 27 (H3K27me3), promotes chromatin condensation, leading to a reduction of gene transcription. EZH2 mutations/misregulation caused a reduction in H3K27me3, which results in altered transcriptional programs and malignant transformation in several cancers [85]. When MM cells are cultured in the presence of BMSCs or fibronectin an increase in the inactivating phosphorylation of S21 of the methyl transferase EZH2 was observed, leading to uncondensed chromatin due to reduced methylation of histone H3. Doxorubicin treatment induces a reduction of S21 inactivating phosphorylation of EZH2 in MM cells cultured alone, leading to methylation of H3 with consequent chromatin condensation, which results in apoptosis. However, the presence of BMSCs or fibronectin prevents H3 methylation by enhancing EZH2 S21 phosphorylation, promoting drug resistance. In particular, the adhesion of MM to BMSCs determines hypomethylation of H3 in the promoter regions of the antiapoptotic *IGF-1* and *Bcl2* genes, resulting in their transcription, with the consequent induction of drug resistance [84].

In conclusion, there is compelling evidence that the BM microenvironment-mediated expression of several TFs, such as c-MYC, JunB and c-MAF, and the modulation of chromatin condensation can mediate drug resistance in MM, envisioning a possible targeting of these molecules in the anti-MM armamentarium.

## 4. miRNA Expression is Modulated by the Bone Marrow Microenvironment and Promotes Plasma Cell Drug Resistance

**Figure 4:**
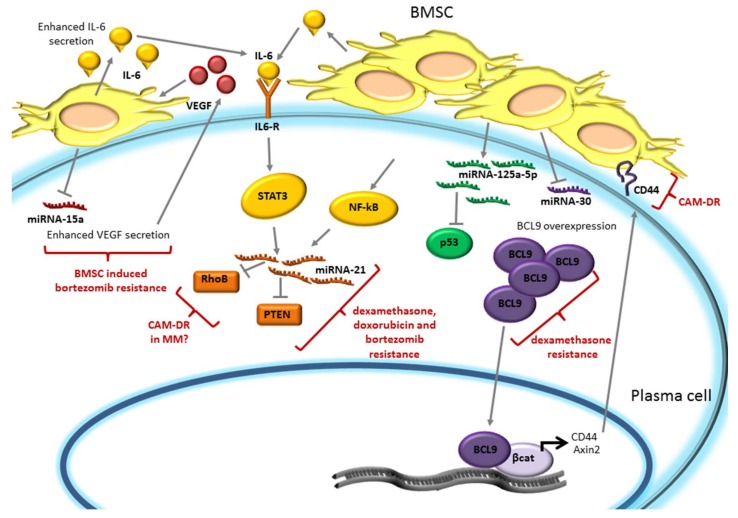
Drug response is modulated by Bone Marrow (BM) microenvironment dependent regulation of miRNAs. Bone Marrow Stromal Cells (BMSCs) may promote bortezomib, dexamethasone, or doxorubicin drug resistance by reducing miRNA-15a and miRNA-30 expression and by upregulating miRNA-21 and miRNA-125a-5p levels. In particular, the reduction of miRNA-15a enhances Vascular Endothelial Growth Factor (VEGF) secretion, promoting bortezomib resistance. Dexamethasone, doxorubicin, and bortezomib resistance could be mediated by STAT3- or Nuclear Factor kappa-light-chain-enhancer of activated B cells NF-κB-dependent upregulation of miRNA-21. BMSCs inhibit p53 by upregulating miRNA-125a-5p and could mediate dexamethasone resistance through BCL9 overexpression due to miRNA-30 downmodulation. In turn, BCL9-induced CD44 expression reinforces Cell Adhesion Mediated–Drug Resistance (CAM-DR). STAT3: Signal transducer and activator of transcription 3; BCL9: B Cell CLL/Lymphoma 9; RhoB: Ras homolog gene family, member B; PTEN: Phosphatase and Tensin homolog.

The cross-talk between the bone marrow microenvironment and MM cells also involves several miRNAs. miRNAs are short, single-strand, non-coding RNAs (19–25 nt) that target the 3′-untranslated region (3′-UTR) of mRNAs preventing protein translation and, therefore, regulating proliferation, metabolism, aging, and cell death [86,87]. BMSCs-MM interaction has been shown to modulate the expression of several miRNAs in MM cells (Figure 4).

miRNA-15a is associated with G1/S arrest and blocking of proliferation [88]. It has been demonstrated that miRNA-15a expression was enhanced in MM cell lines U-266 and H929 when treated with bortezomib, however, this bortezomib-induced miRNA-15a upregulation was suppressed when MM cells were co-cultured with BMSCs, promoting bortezomib-induced drug resistance [88]. Since VEGF is a target of miRNA-15a [89,90], its secretion is enhanced by miRNA-15a downregulation. VEGF, in turn, stimulates IL-6 secretion by BMSCs, providing a favorable microenvironment niche for MM cells proliferation. Therefore, via suppressing bortezomib dependent miRNA-15a upregulation, BMSCs protect MM cells from bortezomib-induced cytotoxicity and support MM cells survival [88].

miRNA-21 was shown to have an oncogenic role in several cancers [91,92,93] and also in MM it is expressed and implicated in malignant development [94]. Modulation of miRNA-21 expression in MM cells affects drug response: miRNA-21 upregulation reduced dexamethasone-, doxorubicine-, and bortezomib-induced apoptosis, while its inhibition sensitizes MM cells to dexamethasone and doxorubicine [95]. miRNA-21 overexpression induced a downregulation of its targets, such as the tumor suppressor phosphatase and tensin homolog (PTEN), promoting MM cell viability [96], and of RhoB, a factor overexpressed in MM that is involved in adhesion-mediated drug resistance (CAM-DR) in other cancer types [97]. It was shown that the adhesion of MM to BMSCs increased miRNA-21 expression through the NF-κB pathway, since the treatment with BAY (an NF-κB specific inhibitor) or with bortezomib (that inhibits NF-κB pathway) prevented miRNA-21 upregulation in MM cells [95]. Moreover miRNA-21 expression was also induced by the IL-6/STAT3 axis [98]. Therefore, the BM microenvironment, through IL-6 or MM-BMSCs adhesion, could protect from drug-induced cytotoxicity, through the upregulation of miRNA-21.

miR-125a-5p is highly expressed in patients carrying the t(4;14) translocation, thus belonging to the TC4 group of TC (Translocation/Cyclin D) prognostic classification [99]. In MM miR-125a-5p was shown to downregulate the p53 pathway, since its expression in H929 cells induced a reduction of p53 mRNA and protein levels and of its downstream targets, such as cyclin-dependent kinase inhibitor 1A CDKN1A/p21, Bcl2 Associated X BAX, Growth Arrest And DNA-Damage-Inducible 45 Alpha (GADD45A), and Mouse Double Minutes 2 (MDM2). Oppositely, its inhibition in MM1S cells increased levels of both p53 and of its targets. The adhesion of MM cells with BMSCs or HS-5 stromal cells enhanced miRNA-125a-5p expression with the consequent reduction of p53 levels, suggesting that this miRNA may be involved in growth-promoting activity of BMSCs on MM cells. Importantly, its inhibition induced a reduction of viability of MM cells also in the presence of BMSCs [100]. Since it is well established that p53 inactivation could mediate drug resistance, it is likely that BMSC mediated increased miRNA-125a-5p, which could modulate drug sensitivity of MM cells. Indeed, in leukemia cells, an association between miRNA-125a and daunorubicin resistance was demonstrated [101]. However, further investigation will be required to definitely demonstrate a role of miRNA-125a-5p in drug resistance in MM.

The miR-30 family is downregulated in most MM patients compared to normal PCs. In particular, miR-30c shows the lowest level of expression in MM patient samples and the higher expression level in normal PCs [102]. This family of miRNAs was demonstrated to control BCL9, a coactivator of β-catenin. Ectopic expression of the miR-30 family in the H929 MM cell line, and in particular of miR30c, was associated with a significant reduction in the expression of BCL9 mRNA and protein, and of its transcriptional targets Axin2 and CD44. MM-HS-5 stromal cell co-culture reduced miRNA-30 family expression with the consequent BCL9, Axin2, and CD44 upregulation. Conversely, ectopic expression of miR-30 in H929 co-cultured with stromal cells, restored MM cell sensitivity to dexamethasone, suggesting that the BM microenvironment can also promote drug resistance of PCs through miRNA-30 downmodulation [102]. Moreover, the BCL9 transcriptional target CD44, enhancing BMSCs-MM cell adhesion, was previously demonstrated to be a component of CAM-DR [103].

In summary, the BM microenvironment dependent fine regulation of several miRNAs can sustain PC survival, mediating bortezomib, dexamethasone, or doxorubicin resistance.

## 5. Conclusions

The evidence described above further supports the outlook whereby MM is an extremely complex disease that displays a recurrent drug-resistant phenotype as long as the disease progresses after sequential waves of therapies. The bone marrow microenvironment is central to this process in that it also changes with disease evolution. Thus, as much as for mapping the genetic evolution of plasma cellular clone(s), it is crucial to describe the serial modifications that the MM milieu accumulates, which likely greatly contributes to the sustenance of the drug-resistant phenotype. An intricate intermingling of events originating in malignant plasma cells, in the BM surrounding cells and in the extracellular space, creates a microcosm, in which MM cells find a super-favorable *pabulum* for their continuous evolution. The identification of vulnerabilities in such a tumor environment is central to rationalizing actual and future treatments. Further research will have to test if a multilayered therapeutic approach, which targets fitness-promoting pathways, transcriptional rewiring, protection from autophagy, deregulation of non-coding RNAs, and other mechanisms, will finally overcome the elusive nature of MM. The final goal is to hamper the ultimate resources malignant plasma cells rely onto to persist in the BM or other niches, and that eventually lead to the recurrence of the disease.

## Abbreviations

3-MA	3-Methyladenine
4-OHT	4-hydroxytamoxifen
ATF4	transcription factor 4
ATF6	Activating Transcription Factor-6
Atg3	Atrogin 3
Atg5	Atrogin 5
Atg7	Atrogin 7
BAX	Bcl2 Associated X
Bcl2	B-cell lymphoma 2
BCl9	B Cell CLL/Lymphoma 9
Bcl-XL	B-cell lymphoma-extra large
BM	Bone Marrow
BMP6	Bone Morphogenetic Protein 6
BMSC	Bone Marrow Stromal Cells
CAFs	Cancer Associated Fibroblasts
CAM-DR	cell adhesion-mediated drug resistance
CD47	Cluster of Differentiation 47
CDKN1A/p21	cyclin dependent kinase inhibitor 1A
CHOP	C/EBP-homologous protein
c-MAF	Avian Muscoloaponeurotic Fibrosarcoma
CQ	Chloroquine
eIF2α	eukaryotic translation initiation factor 2α
ER	Endoplasmic Reticulum
ERAD	ER-associated degradation
EZH2	Enhancer of Zeste Homolog 2
FAM46C	Family with Sequence Similarity 46 Member C
FGF2	Fibroblast Growth Factor 2
FOXO3	Forkhead Box O3
GADD54A	Growth Arrest and DNA-Damage Inducible 45 Alpha
GC	Glucocorticoid
Grp78/Bip	Bglucose regulated protein 78/Binding immunoglobulin protein
GSK3β	Glycogen synthase kinase 3β
H3K27me3	Histone H3 trimethylated in Lysine 27
HCC	Hepatocellular carcinoma
HIF1α	Hypoxia-inducible factor 1α
HSP70	Heat Shock Protein 70
HSPs	Heat shock chaperone proteins
IGF-1	Insulin Growth Factor 1
IL-6	Interleukin-6
IL-8	Interleukin-8
IRE1α	Inositol Requiring Enzyme 1
LC3	microtubule-associated protein 1A/1B-light chain 3
Mcl1	Myeloid cell leukemia 1
MDM2	Mouse Double Minute 2
MEK/MAPK	Mitogen-activated protein kinase kinase/mitogen-activated protein kinase
MGUS	Monoclonal Gammopathy of Undetermined Significance
MM	Multiple Myeloma
mTOR	mammalian target of rapamycine
MYC	V-Myc Avian Myelocytomatosis Viral Oncogene
MYC T-ALL	MYC-induced T cell acute lymphoblastic leukemia
NF-ĸB	Nuclear Factor kappa-light-chain-enhancer of activated B cells
Nrf2	nuclear factor-erythroid 2-related factor 2
PD-L1	Programmed death-ligand 1
PERK	kinase PKR-like ER kinase
PIs	Proteasome inhibitors
POMP	Proteasome maturation proteins
PTEN	Phosphate and tensin homolog
RhoB	Ras homolog gene family, member B
RIDD	IRE1α-dependent decay process
ROS	Reactive Oxygen Species
ROS	Reactive Oxigen Species
sMM	Smouldering Myeloma
Sor	Sorafenib
SQSTM1/p62	Sequestosome-1/p62
STAT3	Signal transducer and activator of transcription 3
TFs	Transcription factors
TGFβ	Tumor Growth Factor-β
UPS	Ubiquitin Proteasome System
VEGF	Vascular Endothelial Growth Factor
XBP1	X-box Binding Protein 1
